# Carcass characteristics, meat quality, sensory palatability and chemical composition of Thai native cattle grazing in lowland and Phu Phan mountain forest

**DOI:** 10.5713/ab.23.0265

**Published:** 2023-11-01

**Authors:** Nirawan Gunun, Chatchai Kaewpila, Rattikan Suwannasing, Waroon Khota, Pichad Khejornsart, Chirasak Phoemchalard, Norakamol Laorodphan, Piyawit Kesorn, Pongsatorn Gunun

**Affiliations:** 1 Department of Animal Science, Faculty of Technology, Udon Thani Rajabhat University, Udon Thani 41000, Thailand; 2 Department of Animal Science, Faculty of Natural Resources, Rajamangala University of Technology Isan, Sakon Nakhon Campus, Phangkhon, Sakon Nakhon 47160, Thailand; 3 Department of Agriculture and Resources, Faculty of Natural Resources and Agro-Industry, Kasetsart University Chalermphrakiat Sakon Nakhon Province Campus, Sakon Nakhon 47000, Thailand; 4 Department of Agriculture, Mahidol University, Amnatcharoen Campus, Amnatcharoen 37000, Thailand; 5 Animal Science and Aquaculture Program, Faculty of Food and Agricultural Technology, Pibulsongkram Rajabhat University, Phitsanulok 65000, Thailand

**Keywords:** Cattle, Chemical Composition, Fatty Acid Profiles, Grazing Systems, Meat Quality, Sensory Evaluation

## Abstract

**Objective:**

The aim of this study was to assess the effect of Thai native cattle grazing in the lowland or mountain forest on carcass characteristics, meat quality, sensory palatability, and chemical composition.

**Methods:**

Twelve male Thai native cattle with an average weight of 110±10 kg are allowed to be grazing in the lowland or Phu Phan mountain forest during the rainy season in northeastern Thailand.

**Results:**

The carcass characteristics, meat pH, and meat color were unaffected by treatment (p>0.05). The boiling loss was lower in the cattle grazing on the mountain forest (p = 0.027). The cattle grazing in the mountain forest had increased shear force (p = 0.039), tenderness (p = 0.011), and flavor intensity (p = 0.003). The protein and fat were higher (p<0.001 and p = 0.035, respectively) in cattle grazing in the mountain forest. The different grazing systems of the cattle had no effect (p>0.05) fatty acids in meat, except for capric acid (C10:0) and lauric acid (C12:0), which were higher (p = 0.046 and p = 0.049, respectively) when the cattle were grazing in the mountain forest. The different grazing systems did not influence (p>0.05) the unsaturated fatty acids in meat.

**Conclusion:**

Thai native cattle grazing in the Phu Phan mountain forest in the rainy season improves meat quality, sensory evaluation, and chemical composition.

## INTRODUCTION

Throughout the past century, the native beef cattle production system has played an important role in sustaining households and communities [1]. The *Bos indicus* (Zebu) species of cattle that are native to Thailand are distinguishable by their humped backs [2]. There are four indigenous breeds named Khaolumpoon cattle (located in northern Thailand), Isaan cattle (located in northeastern Thailand), Lan cattle (located in central Thailand), and Chon cattle (located in southern Thailand) [3]. Their small body size (adult females weigh 200 to 270 kg and males 300 to 350 kg) is accompanied by high fertility, heat tolerance, resistance to ectoparasites, and well-adaptedness to the tropical environment and climbing mountains [2,4]. Traditional Isaan cattle are taken to open markets with an undefined meat grade for beef production. Consumers prefer to use local native cattle to cook laab or koi (spicy minced beef salad), which are local foods in the northeast of Thailand. The rice-livestock integration system combines rice cultivation with cattle raising, resulting in a mutually beneficial relationship between the two sectors [5]. During the dry season, farmers rear cattle for grazing in rice fields following rice postharvest, whereas during the rainy season, they use them for rice production. Additionally, there has been a reduction in the usable grazing land for cattle [2]. Hence, farmers raise cattle in natural grasslands and contain them in pens with fresh-cut grass or raise them in the mountains [1].

Meat contains essential nutrients (protein, carbohydrate, fat, essential amino acids, minerals, vitamins, etc.). Currently, the number of consumers interested in food safety, health products, and environmental impacts is growing steadily [6]. Consequently, the food marketing industry devotes considerable resources to enhancing food’s healthfulness [7,8]. Strategies to improve meat quality, animal welfare and health, rural development, carbon footprint, and the biodiversity of grasslands are becoming more important to consumers [9]. In mountainous regions of Thailand, they provide forage for animals grazing during the rainy season. Gunun et al [10] found that Isaan cattle are grazing 41 species plants found in Phu Phan mountain, Sakon Nakhon, Thailand, during the the rainy season. These are mainly pek (*Vietnamosasa pusilla*) and other plants, including native grass, trees, shrubs, Thai herbs, annuals, and climers. In addition, the crude protein (CP), ether extract (EE), and gross energy of plants were between 3.5% to 26.3%, 0.6% to 6.7%, and 3,103.1 to 5,488.8 kcal/kg, respectively. Duanyai et al [11] reported that cattle were grazing 37 species plants found in the mountain forest during the rainy season in Ubon Ratchathani, Thailand. Uriyapongsan et al [12] found that Isaan cattle selectively grazed pek, *Leucaena leucocephala*, *Brachiaria mutica*, and nine species of native grass in the rainy season of lowland close to Ubolratana Dam, Khon Kaen, Thailand. Furthermore, in the lowlands, especially close to the dam, cattle continuously graze the pasture and also limit plant growth, resulting in lower grass production and nutritive value. This may lower the nutrient requirements, growth performance, meat quality, and chemical composition of the cattle. Cattle graze forage during the rainy season (May to October) in the mountain forest, while after rice harvest and the dry season, farmers raise cattle in the rice fields, resulting in higher plant growth, plant species richness, and nutritive value in the mountain forest [12,13].

Previous studies reported that grazing in the mountain, organic rice field, or pasture of cattle did not alter meat characteristics or chemical composition, while conjugated linoleic acid (CLA) and selenium levels in the meat were higher when the cattle were grazing in the mountain forest [11]. Furthermore, non-pesticide residues and antibiotics are present when cattle are grazing under different feeding systems. Ådnøy et al [14] reported that the Norwegian lambs grazing mountain pastures improved the meat tenderness and chemical composition, including protein, ash, and polyunsaturated fatty acids (PUFA), when compared with lowland pastures.

However, no studies of Thai native cattle grazing in the mountain forest and lowland have been conducted. We hypothesize that Thai native cattle grazing in the Phu Phan mountain forest improves meat quality, sensory evaluation, and chemical composition. As a result, the purpose of this research study was to assess the influence of two grazing systems (lowland and mountain forest) during the rainy season on carcass characteristics, meat quality, sensory evaluation, and chemical composition in Thai native cattle.

## MATERIALS AND METHODS

### Animal care

The present research study was approved by the Rajamangala University of Technology Isan for both animal care and experimental procedures (approval number 8/2564).

### Experimental grazing systems

Twelve male Esaan cattle with an average weight of 110± 10 kg were then split into two equal groups and allowed to use two grazing systems for a 170-day experiment (May to October 2021) during the rainy season in Thailand. The treatments were as follows: lowland (Pannanikom, Sakon Nakhon, Thailand, 17°16′16″N, 103°45′43″E) at 180 to 200 m asl, the cattle grazing natural grassland close to the Nam Un Dam at daytime (the area of 500 hectare) and keeping cattle in house all night; and mountain forest (Pannanikom, Sakon Nakhon, Thailand, 17°15′38″N, 103°48′40″E) at 250 to 400 m asl, the cattle’s availability of grazing in the forest of Phu Phan mountain (the area of 800 hectare). Isaan cattle lives on this mountain all the time during the experiment.

### Data collection and sampling procedures

All the cattle were fasted for 12 hours and randomly assigned to be slaughtered at the Sakon Nakhon municipality slaughterhouse in Sakon Nakhon, Thailand. All experimental methods followed the Department of Livestock Development, Ministry of Agriculture and Cooperatives, Royal Thai Government’s guidelines for animal welfare. Following slaughter, the hot carcass weight and retail cuts were determined. Image evaluation was performed to evaluate the loin eye area between the 12th and 13th ribs. The removal of *Longissimus lumborum* was achieved by cutting it out of the loin muscles located between the 12th and 13th rib on every carcass’s right side. These samples were used to assess various aspects of meat, including its pH level, water holding capacity (WHC), color, shear force, sensory analysis, chemical composition, and fatty acid profiles.

Calibrating with two buffer solutions was done before measuring the meat pH. The first buffer, which was neutral, had a pH scale reading of 7; the second one had a lower reading of 4. Evaluation of meat pH levels was taken at 45 minutes postmortem (pH_45_) and 28 days postmortem (pH_u_) using a portable pH meter (FiveGo, Mettler-Toledo GmbH, Greifensee, Switzerland) in triplicates. In order to analyze meat color effectively and accurately [15], the Minolta Chroma Meter (CR-300, Osaka, Japan) was calibrated first using a standard white tile. An illuminant D65, a measuring area of 8 mm, and a viewing angle of 0° were used for this measurement. To prepare meat samples for color measurement, they were sliced and allowed to rest for an 1 hour before being analyzed. Three measurements were taken for the Commission Internationale de l’Eclairage (CIE) L*a*b* colors.

The Honikel [16] method was used to estimate drip loss, while boiling and thawing losses were assessed through the slicing of 2.5 cm beef samples and their placement in plastic bags. Boiling loss was determined by boiling the samples in an 80°C water bath until an internal temperature of 70°C was reached. Additionally, a meat sample was frozen at −20°C for 24 hours prior to being thawed at 4°C for another 24 hours. The percentage of drip, boiling, and thawing losses was determined by assessing the weight difference before and after refrigerating, boiling, and thawing. The meat from the boiling loss study was cut using a steel hollowcore device with a diameter of 1.27 cm to measure shear force [17] with a TA XT2 Texture Analyzer (Texture Technologies Corp., Scarsdale, NY, USA). Thirty students and teachers from the Faculty of Natural Resources at Rajamangala University of Technology Isan, Sakon Nakhon Campus, who had been trained in sensory evaluation using the method of Viriyajare [18], were chosen to be on the test panel. Meat samples have been analyzed for their moisture, protein, fat, and ash content [19]. Finally, meat fatty acid profiles were determined using gas chromatography (GC 8890; Agilent Technologies Ltd., Santa Clara County, CA, USA) [20].

### Statistical analysis

All data were analyzed using SAS version 6.12 software, and PROC TTEST was used to compare cattle grazing in lowland and mountain forest [21]. At p<0.05, statistical significance was evaluated.

## RESULTS

### Carcass charateristics

The slaugher weight, hot carcass, dressing percentage, and loin eye area were similar among groups (p>0.05) (Table 1). There was no effect of treatment on retail cut percentage or other organs (p>0.05). The spleen was increased (p = 0.003) from cattle grazing in the mountain forest, while the abomasum was increased (p<0.001) from cattle grazing in lowland.

### Meat quality

The different grazing systems of the cattle had no effect on meat pH (p>0.05) (Table 2). The drip loss, thawing loss, and L*, a*, or b* of the meat color were similar among treatments (p>0.05), but the cattle grazing in the mountain forest had a lower boiling loss (p = 0.027). The shear force, tenderness, and flavorness were increased (p<0.039, p = 0.011, and p = 0.003, respectively) in cattle grazing in the mountain forest (Table 3), while the juiciness and overall acceptability were not affected (p>0.05) when the cattle were grazing in different systems. Protein and fat in the meat were increased when the cattle were grazing in the mountain forest (p<0.001 and p = 0.035, respectively).

### Meat fatty acid profiles

The different grazing systems of the cattle did not affect (p> 0.05) saturated fatty acids (SFA) in meat, except for C10:0 and C12:0, which were increased (p = 0.046 and p = 0.049, respectively) when the cattle were grazing in the mountain (Table 4). The unsaturated fatty acids (UFA), including C18:1 cis-9 + tran-9, C18:2 cis-9,12 + tran-9,12, and C18:3 cis-9, 12,15, had similar results among treatments (p>0.05).

## DISCUSSION

### Carcass charateristics

The average slaughter weight and dressing percentage of the Thai native cattle grazing in lowland and mountainous areas in the rainy season were 161.34 kg and 48.61%, respectively. Similarly, Uriyapongsan et al [12] reported that the Isaan cattle grazing in northeastern Thailand in the rainy season had slaughter weight and dressing percentages of 164.70 kg and 46.70%, respectively. However, Uriyapongsan et al.’s [12] report of the loin eye area at 41.20 cm^2^ was lower than the loin eye area (54.08 cm^2^) of the cattle in our study during the rainy season. These results might be due to different areas of study, geography, types of forage, feeding, age, sex, etc.

The spleen is the body’s immune organ and is responsible for initiating immune responses to antigens in the blood, producing antibodies, and filtering the blood of damaged red blood cells and microbes [22,23]. Meyer et al [24] discovered that larger spleen sizes influence feed utilization efficiency in sheep. The cattle spleen mass increased with grazing in the mountain forest in our study; it is plausible that the size of the spleen may increase in response to infection and utilize feed more efficiently. The different grazing systems of the cattle had no effect on the rumen, reticulum, and omasum, while the abomasum was larger when the cattle were grazing in lowland. However, Duanyai et al [11] reported that crossbred cattle (Brahman×Thai native) grazing in the mountain forest, organic rice field, and pasture had similar rumen, reticulum, omasum, and abomasum. It is unclear why the effects of higher abomasum mass in the cattle lowland group were studied.

### Meat quality

The meat pH at 45 min and 28 days in *Longissimus lumborum* in Thai native cattle was in the normal range when compared to previous reports [25,26]. Whether the cattle were grazing in mountain forest or lowland had no effect on the meat pH. Similarly, Ådnøy et al [14] found that lambs grazing in mountain and lowland did not change meat pH. The WHC of meat products is an essential factor that influences product yield, which has economic ramifications and affects eating quality [27]. The ability of meat to retain water is a crucial quality characteristic that impacts its color, juiciness, and tenderness. Factors influencing the WHC of meat include the animal’s genotype, diet, muscle properties, proteolytic activity, pre-slaughter stress, post-slaughter management, aging, cooking, cooling methods, etc. [27,28]. In the current study, boiling loss was lowered in more active mountain forest cattle due to an increase in connective tissue, as shown by higher shear force values. When compared to the same breeds, the boiling loss observed in this study was in line with the findings of Phoemchalard et al [29]’s study but lower than that of Thai native cattle fattened on grass or grass-legume pastures [25]. In fact, the presence of proteolytic enzymes can break down muscle proteins and reduce their ability to hold water, while meat with a higher collagen content tends to have a lower WHC because the collagen fibers can bind to water molecules and reduce their availability to the muscle proteins [30,31].

As described by Belew et al [32], the mountain forest grazing cattle in our study had tougher meat (shear force = 65.07 N), and due to cultural eating preferences, consumers preferred this tougher meat to the intermediately tender meat (shear force = 36.36 N) of lowland grazing cattle. The average shear force presented in this study was consistent with previous reports [25,29] in Thai native and/or crossbred Brahman×Thai native cattle. In addition, the grazing in the mountain forest enhanced the meat’s tenderness and flavor when compared with those grazing in the lowland. These results agree with Ådnøy et al [14], who indicated that the meat tenderness was higher in the lambs grazing in mountain pastures than in lowland pastures. A particular level of fat has been proposed to be needed to produce high-quality meat, as fat is frequently related to sensory evaluation. According to O’Quinn et al [33], higher fat content would cause greater increases in the tenderness and flavor scores of cattle meat. These results might be due to the mountain cattle’s enhanced fat content in meat, which is related to higher tenderness and flavor.

Cattle grazing in the mountain forest have a higher fat content in their meat. In contrast, Ådnøy et al [14] reported that lambs grazing on mountain pastures in Norway had a reduced fat content compared to lambs grazing on lowland pastures. Issan cattle grazing 12 species of plants, mainly native grass, contain 0.64% to 3.34% EE in the rowland close to Ubolratana Dam, northeastern Thailand [12]. Gunun et al [10] found that when cattle were grazing in the forest of the Phu Phan mountain in northeastern Thailand, they had access to 41 plant species, and 92% of the plants contained 1.4% to 6.7% EE. These results could be due to cattle grazing on plants with higher EE content in the mountain than in the lowland, resulting in increased fat content in meat. Moreover, mountain forest cattle have a higher protein content in their meat, which is consistent with the results reported by Ådnøy et al [14], who found the lambs grazing in the mountain pastures had a higher protein content in their meat than those grazing in lowland. The Isaan cattle grazing plant leaves of 23 species containing 10.1% to 26.3% CP in the forest of the Phu Phan mountain [10], while Uriyapongsan et al [12] found cattle grazing plant leaves of only *L. leucocephala* in the lowland. This makes it plausible that the cattle in mountains graze many plant leaf species and are not grazing grass, which also enhances protein content in meat.

### Meat fatty acid profiles

Beef muscle fatty acid accumulation is dependent on dietary fatty acid composition, and rumen microbial biohydrogenation [34,35]. The higher C10:0 and C12:0 in the meat of the cattle were grazing in the mountain forest. Krabok (*Irvingia malayana*, Oliv. ex. A. Benn.) fruit falls to the ground during October in the late rainy season in the Phu Phan mountain, Thailand. According to the survey in our study, the most cattle selectively graze krobok fruit than other feed in the mountain forest. Krabok seed rich in C10:0 and C12:0 consists of 1.6% and 42.0%, respectively [36]. The increased C10:0 and C12:0 in meat indicated that the cattle were mostly selectively grazing krabok fruit from the groud in the late rainy season. Another reason is that the plant diversity in the cattle’s selective grazing in the mountain forest may result in higher SFA levels, especially C10:0 and C12:0, and their accumulation in meat.

The most prevalent fatty acids in *Longgissimus lumborum* of Thai native cattle were palmitic acid (C16:0; 29.48% to 30.39%), which was similar, while stearic acid (C18:0; 29.48% to 30.39%) was higher and oleic acid (C18:1; 27.06% to 27.49%) was lower when compared with Jaturasitha et al [25]. In their reports, the cattle were grazing guinea grass (*Panicum maxima*) or guinea grass-legume (*Stylosanthes guianensis*) pastures. It is possible that differences in age, grazing systems, and type of forage may affect the fatty acid composition. The various grazing systems did not alter C18:1, linoleic acid (C18:2), or linolenic acid (C18:3) in the meat of cattle. The results agree with the previous studies report that here is no effect of cattle grazing different grass and legume pastures on C18:1, C18:2, or C18:3 in meat [25,37]. Saturated fatty acids, and UFA, including monounsaturated fatty acids (MUFA) and PUFA were not altered among treatments. It indicated that the Thai native beef cattle grazing different types of grass and plants in various grazing systems did not influence their fatty acid profile. Contrast that with Ådnøy et al [14], who reported that lambs grazing in mountain pastures have reduced levels of MUFA and higher levels of PUFA in their meat compared to lambs grazing on lowland pastures. The different results from previous studies may have effects on ruminant genus, grazing behavior, land use, plant varieties, the environment, topography, and other factors. The average SFA and UFA were 67.64% and 32.35%, respectively, in the cattle grazing in lowland and mountain areas, while SFA was higher and UFA was lower than the previous studies [34,38], which reported that they used a total mixed ration and separate feeding (concentrate with paragrass [*Brachiaria mutica*]) and the cattle were reared in separate pens. This could be due to the different feeding systems, the ingredients used in the diet, etc., which have an effect on the fatty acids in meat.

## CONCLUSION

The two grazing systems (lowland and mountain forest) of Thai native cattle did not affect the carcass characteristics, meat color, or fatty acid profile of the meat. Cattle grazing in the mountain forest had decreased boiling loss while increased sensory ratings (tenderness and flavor) and chemical compositions (protein and fat). This suggests that Thai native cattle grazing in the Phu Phan mountain forest during the rainy season improves sensory palatability, or chemical composition, while reducing boiling loss in meat.

## Figures and Tables

**Table 1:** Effects of different grazing systems on carcass characteristics in Thai native cattle

Item	Lowland	Mountain forest	p-value
Slaughter weight (kg)	158.67±13.60	164.00±33.36	0.705
Hot carcass weight (kg)	76.40±6.21	80.19±18.04	0.636
Dressing percentage	48.18±1.64	49.04±6.86	0.775
Loin eye area (cm^2^)	55.34±2.73	52.83±3.54	0.722
Retail cut (%)			
Brisket	1.69±0.12	2.07±0.43	0.070
Filet	2.17±0.12	2.05±0.39	0.539
Flank	4.42±0.26	4.74±0.87	0.532
* * *Longissimus thoracis et lumborum*	7.58±0.64	6.43±1.29	0.079
Visceral fat	1.25±0.65	2.60±1.35	0.053
Offal (%)			
Heart	0.28±0.02	0.32±0.03	0.102
Liver	0.50±0.09	0.45 ± 0.04	0.380
Lung	1.19±0.26	1.03±0.17	0.230
Spleen	0.22±0.05	0.34±0.08	0.003
Kidney	0.20±0.02	0.19±0.02	0.645
Reticulum	0.43±0.08	0.42±0.07	0.944
Rumen	1.95±0.22	2.14±0.31	0.257
Omasum	0.74±0.11	0.75±0.06	0.877
Abomasum	0.48±0.04	0.34±0.03	<0.001
Small intestine	1.15±0.16	1.23±0.07	0.329
Large intestine	0.92±0.20	0.84±0.16	0.430
Bile	0.11±0.03	0.08±0.03	0.207
Intestinal secretion	0.83±0.45	1.08±0.56	0.499
Other organs (%)			
Head and horn	6.37±0.56	5.91±0.19	0.108
Skin	8.32±0.79	8.74±0.90	0.405
Tail	0.44±0.06	0.57±0.33	0.341
Penis	0.63±0.06	0.68±0.10	0.418
Tongue	0.42±0.07	0.52±0.17	0.231

**Table 2:** Effects of different grazing systems on meat quality in Thai native cattle

Item	Lowland	Mountain forest	p-value
pH 45 min (pH_45_)	6.96±0.46	6.88±0.25	0.737
pH 28 days (pH_u_)	5.97±0.38	5.76±0.26	0.293
Water holding capacity (WHC, %)			
Drip loss	9.10±2.91	8.76±3.51	0.857
Thawing loss	11.09±3.44	6.50±4.54	0.077
Boiling loss	34.42±6.99	23.94±7.08	0.027
Meat colour			
Lightness (L*)	34.26±5.31	35.63±3.29	0.602
Redness (a*)	13.36±1.48	13.52±3.24	0.917
Yellowness (b*)	13.66±2.06	14.17±2.29	0.691

**Table 3:** Effects of different grazing systems on shear force, sensory properties, and chemical composition in Thai native cattle

Item	Lowland	Mountain forest	p-value
Shear force, N	36.36±16.06	65.07±25.04	0.039
Sensory ratings			
Tenderness	6.75±0.32	7.21±0.15	0.011
Juiciness	6.41±0.43	6.57±0.32	0.498
Flavourness	6.87±0.53	7.34±0.14	0.003
Overall acceptability	6.79±0.35	7.05±0.32	0.216
Chemical composition (%)			
Moisture	74.77±2.27	73.21±0.84	0.146
Protein	12.98±1.42	18.67±1.34	<0.001
Fat	1.56±0.98	2.76±0.47	0.035
Ash	0.93±0.07	0.88±0.11	0.386

Sensory ratings (30 people) 1 = highly unfavorable, 5 = average, 9 = highly favorable.

**Table 4:** Effects of different grazing systems on meat fatty acid profiles in Thai native cattle

Item	Lowland	Mountain forest	p-value
C8:0	0.15±0.12	0.04±0.02	0.090
C10:0	0.04±0.01	0.06±0.01	0.046
C12:0	0.07±0.01	0.09±0.01	0.049
C13:0	0.02±0.014	0.02±0.004	1.000
C14:0	3.81±1.25	4.92±0.41	0.098
C14:1 cis-9	0.30±0.12	0.39±0.14	0.303
C15:0	0.92±0.30	0.77±0.10	0.341
C16:0	29.48±3.79	30.39±1.29	0.624
C16:1 cis-9	2.24±0.59	2.10±0.72	0.745
C17:0	1.80±0.24	1.75±0.16	0.684
C18:0	30.17±6.20	29.79±5.96	0.922
C18:1 cis-9 + tran-9	27.49±3.51	27.06±5.19	0.882
C18:2 cis-9,12 + tran-9,12	2.13±1.16	1.39±0.33	0.208
C18:3 cis-9,12,15	0.48±0.25	0.59±0.11	0.433
C20:0	0.23±0.08	0.25±0.08	0.676
C20:1 cis-11	0.14±0.07	0.13±0.01	0.648
C20:5 cis-5,8,11,14,17	0.20±0.14	0.08±0.04	0.105
C22:0	0.05±0.09	0.04±0.02	0.780
C23:0	0.27±0.23	0.15±0.06	0.291
Saturated fatty acids (SFA)	67.02±2.55	68.27±5.78	0.668
Unsaturated fatty acids (UFA)	32.98±2.55	31.73±5.78	0.669
Monounsaturated fatty acids (MUFA)	30.17±3.84	29.67±6.00	0.879
Polyunsaturated fatty acids (PUFA)	2.81±1.52	2.05±0.44	0.315
